# Alcohol-induced decrease in muscle protein synthesis associated with increased binding of mTOR and raptor: Comparable effects in young and mature rats

**DOI:** 10.1186/1743-7075-6-4

**Published:** 2009-01-20

**Authors:** Charles H Lang, Anne M Pruznak, Gerald J Nystrom, Thomas C Vary

**Affiliations:** 1 Department of Cellular and Molecular Physiology, The Pennsylvania State University College of Medicine, Hershey, PA 17033, USA

## Abstract

**Background:**

Acute alcohol (EtOH) intoxication decreases muscle protein synthesis via inhibition of mTOR-dependent translation initiation. However, these studies have been performed in relatively young rapidly growing rats in which muscle protein accretion is more sensitive to growth factor and nutrient stimulation. Furthermore, some in vivo-produced effects of EtOH vary in an age-dependent manner. The hypothesis tested in the present study was that young rats will show a more pronounced decrement in muscle protein synthesis than older mature rats in response to acute EtOH intoxication.

**Methods:**

Male F344 rats were studied at approximately 3 (young) or 12 (mature) months of age. Young rats were injected intraperitoneally with 75 mmol/kg of EtOH, and mature rats injected with either 75 or 90 mmol/kg EtOH. Time-matched saline-injected control rats were included for both age groups. Gastrocnemius protein synthesis and the activity of the mTOR pathway were assessed 2.5 h after EtOH using [^3^H]-labeled phenylalanine and the phosphorylation of various protein factors known to regulate peptide-chain initiation.

**Results:**

Blood alcohol levels (BALs) were lower in mature rats compared to young rats after administration of 75 mmol/kg EtOH (154 ± 23 vs 265 ± 24 mg/dL). However, injection of 90 mmol/kg EtOH in mature rats produced BALs comparable to that of young rats (281 ± 33 mg/dL). EtOH decreased muscle protein synthesis similarly in both young and high-dose EtOH-treated mature rats. The EtOH-induced changes in both groups were associated with a concomitant reduction in 4E-BP1 phosphorylation, and redistribution of eIF4E between the active eIF4E·eIF4G and inactive eIF4E·4EBP1 complex. Moreover, EtOH increased the binding of mTOR with raptor in a manner which appeared to be AMPK- and TSC-independent. In contrast, although muscle protein synthesis was unchanged in mature rats given low-dose EtOH, compared to control values, the phosphorylation of rpS6 and eIF4G was decreased.

**Conclusion:**

These data indicate that muscle protein synthesis is equally sensitive to the inhibitory effects of EtOH in young rapidly growing rats and older mature rats which are growing more slowly, but that mature rats must be given a relatively larger dose of EtOH to achieve the same BAL. Based on the differential response in mature rats to low- and high-dose EtOH, the decreased protein synthesis was associated with a reduction in mTOR activity which was selectively mediated via a reduction in 4E-BP1 phosphorylation and an increase in mTOR·raptor formation.

## Background

Acute alcohol intoxication decreases muscle protein synthesis in a dose- and time-dependent manner, and this response is largely if not completely abated 24 h after alcohol administration [1,2]. This alcohol-induced decrease is independent of the oxidative metabolism of ethanol and cannot be explained by the over production of either glucocorticoids or selected proinflammatory cytokines, such as tumor necrosis factor (TNF)-α, interleukin (IL)-1 or IL-6 [1,3,4]. However, it is clear that alcohol acutely down regulates translational efficiency which is predominantly mediated by a reduction in peptide-chain initiation [5]. Our previous work indicated this change was independent of altered tyrosine phosphorylation of either the insulin or insulin-like growth factor (IGF)-I receptor, or theronine (Thr)-308 phosphorylation of protein kinase B (PKB; aka Akt) in skeletal muscle [6]. In contrast, our data suggested that the mammalian Target Of Rapamycin (mTOR) played a central role in regulating the alcohol-induced decrease in muscle protein synthesis [1,6,7]. The activity of this serine (Ser)/Thr kinase is most often assessed by phosphorylation of its immediate down stream substrates namely ribosomal protein S6 kinase (S6K1)-1 and the eukaryotic initiation factor 4E (eIF-4E) binding protein-1 (4E-BP1) [8]. In this regard, acute alcohol intoxication decreases the phosphorylation of both S6K1 and 4E-BP1 as well as the autophosphorylation of mTOR itself [1,6,7]. However, the mechanism by which alcohol impairs mTOR activity is poorly defined.

The constitutive or basal rate of muscle protein synthesis is a dynamic process which undergoes marked changes during the life time of the host [9-11]. Muscle protein synthesis rates are elevated in young, rapidly growth animals and then later decrease and reach a relative new steady-state in animals as they mature. However, there remains some controversy regarding whether muscle protein synthesis decreases even further or may actually increase in a compensatory manner in the aged animal or human [12]. Young animals are also especially sensitive to the anabolic actions of growth factors, such as IGF-I and insulin, as well as nutrient signals, such as the branched-chain amino acid leucine [13-15]. Furthermore, in some aspects, young rats appear unusually sensitive to various effects of alcohol [16-18]. Previous investigations of the effect of acute alcohol intoxication on muscle protein synthesis have used relatively young rapidly growing rats and, therefore, it is unknown whether the catabolic effects of alcohol on muscle are also present in older more mature animals.

The present study tests the hypothesis that young rats, which are more sensitive to changes in the prevailing circulating concentrations of growth factors and leucine, will show a more pronounced decrement in muscle protein synthesis in response to acute alcohol intoxication. Moreover, this more precipitous reduction in young rats will be associated with a fall in the content and/or activity (i.e., phosphorylation) of proteins regulating the initiation phase of mRNA translation. As previous studies reported that acute alcohol intoxication does not alter the eIF2/2B system [19], which controls the binding of met-tRNA_i _to the 40S ribosomal subunit to form the 43S preinitiation complex, we therefore focused on elucidating the alcohol-induced changes in a second critical locus of translational regulation involving the binding of the 5'-end of cellular mRNA to the 43S preinitiation complex. In general, this reaction is mediated by the cap-binding protein complex eIF4F which is in turn largely mediated by the kinase activity of mTOR.

## Methods

### Acute alcohol intoxication

Fischer 344/NHsd male rats were obtained from the National Institute on Aging at either 2 months or 11 months of age. Rats were then housed for the next 2–3 weeks at a constant temperature, exposed to a 12:12-h light-dark cycle, and maintained on standard rodent chow (Harlan #2018; Madison, WI) and water ad libitum before experiments were performed. The commercial diet consisted of approximately 18% protein, 6% fat and 3.8% fiber (metabolizable energy 3.3 Kcal/g), and the exact macro- and micro-nutrient composition of the diet is available at . Based on previously described criteria, these groups are considered to represent young adult (3 months) and mature (12 months) for the F344 strain of rat [20]. The current study was *not *designed to examine the effect of aging per se which would necessitate using rats 24–36 months of age. Analysis of data from rats of this older age can be complicated by secondary changes in food consumption, activity levels, and other pathologies which develop during ageing and thereby indirectly affect muscle protein synthesis [21]. The F344 rat strain was used because it is one of the best characterized models of aging in rats and circumvents the large increase in adiposity which develops in the Sprague-Dawley rat with age [22].

Young adult rats were injected intraperitoneally with either ethanol (75 mmol/kg body weight) or an equal volume of 0.9% sterile saline. This ethanol dose was selected because it decreases muscle protein synthesis in Sprague-Dawley rats with body weights between approximately 100–300 g [2,4,6,19]. The protein metabolic effect of ethanol injected intraperitoneal is comparable to an equivalent dose administered by oral gavage [1]. The older mature rats were divided into three groups: *group 1 *– ethanol administered at a dose of 75 mmol/kg; *group 2 *– ethanol administered at a dose of 90 mmol/kg; and *group 3 *– time-matched control rats injected with saline. Two different ethanol doses were used in older mature rats because the blood alcohol concentrations were significantly lower in mature vs young rats when injected with a dose of 75 mmol/kg. Therefore, we included a second group of alcohol-treated mature rats which were injected with a higher dose of ethanol (90 mmol/kg) in order to produce equivalent blood alcohol levels between the young and mature rats. Rats in all groups were fasted overnight prior to the injection of alcohol and food was withheld after alcohol administration. Water was available throughout the experimental protocol.

All experimental protocols involving animals were approved by the Institutional Animal Care and Use Committee of The Pennsylvania State University College of Medicine and adhered to the National Institutes of Health guidelines for the use of experimental animals.

### Muscle protein synthesis

In vivo protein synthesis in gastrocnemius was determined 2.5 h after injection of either alcohol or saline using the flooding-dose technique [23]. Briefly, overnight fasted rats were anesthetized with intraperitoneal pentobarbitol (100 mg/kg) and a catheter placed in the carotid artery. Arterial blood was removed for measuring the plasma concentration of ethanol and various hormones, and then a bolus injection of L- [2,3,4,5,6-^3^H]phenylalanine (Phe; 150 mM, 30 μCi/ml; 1 ml/100 g BW) was injected via the jugular vein. Serial blood samples were drawn at 2, 6 and 10 min after Phe injection for measurement of Phe concentration and radioactivity. Immediately after the final blood sample, the gastrocnemius muscle was excised in its entirety and a portion frozen between aluminum blocks pre-cooled to the temperature of liquid nitrogen and the remaining muscle directly homogenized. Blood was centrifuged and plasma was collected. All tissue and plasma samples were stored at -80°C until analyzed. The frozen muscle was powdered under liquid nitrogen and a portion used to estimate the rate of incorporation of [^3^H]Phe into protein, exactly as described [23].

### Immunoprecipitation and Western blot analysis

The tissue preparation was the same as previously described by our laboratories [1,6,7,19]. Muscle was homogenized in a 1:5 ratio of ice-cold homogenization buffer (pH 7.4) composed of (in mM): 20 mM HEPES, 2 EGTA, 50 NaF, 100 KCl, 0.2 EDTA, 50 β-glycerophosphate, 1 DTT, 0.1 PMSF, 1 benzamidine, 0.5 sodium vanadate, plus one protease inhibitor cocktail tablet from Roche, and clarified by centrifugation. The samples were subjected to SDS-PAGE and the proteins were electrophoretically transferred to PVDF membranes. The blots were incubated with either primary antibodies (unless otherwise noted from Cell Signaling, Beverly, MA) to total (C-20) and Thr1462-phosphorylated TSC2, total 4E-BP1 (Bethyl Laboratories, Montgomery, TX), and total and phosphorylated (Ser1108) eIF4G, total and phosphorylated-S6 (Ser240/Ser244), total and phosphorylated (Thr172) AMP-activated protein kinase (AMPK), as well as total PRAS40 (proline-rich Akt substrate 40; Biosource, Camarillo, CA), GβL (G protein β-subunit-like protein; mLST8) and raptor. In general, blots were washed with TBS-T (1X TBS including 0.1% Tween-20) and incubated with secondary antibody (horseradish peroxidase conjugated goat anti-mouse or goat anti-rabbit IgG) at room temperature. The blots were developed with enhanced chemiluminescence (ECL) Western blotting reagents as per the manufacturer's (Amersham, Piscataway, NJ) instructions. The blots were exposed to X-ray film in a cassette equipped with a DuPont Lightning Plus intensifying screen. After development, the film was scanned (Microtek ScanMaker IV) and analyzed using National Institutes of Health Image 1.6 software.

The eIF4E·4EBP1 and eIF4E·eIF4G complexes were quantified as described [1,6,7,19]. Briefly, eIF4E was immunoprecipitated from aliquots of supernatants using an anti-eIF4E monoclonal antibody (kindly provided by Drs. Jefferson and Kimball; Hershey, PA). Antibody-antigen complexes were collected using magnetic beads, subjected to SDS-PAGE, and proteins transferred to a PVDF membrane. Blots were incubated with a mouse anti-human eIF4E antibody, rabbit anti-rat 4E-BP1 antibody, or rabbit anti-eIF4G antibody.

To maintain potential protein-protein interactions, fresh muscle was also homogenized in CHAPS buffer (pH 7.5) composed of (in mM): 40 HEPES, 120 NaCl, 1 EDTA, 10 pyrophosphate, 10 β-glycerol phosphate, 50 NaF, 1.5 sodium vanadate, 0.3% CHAPS, and 1 protease inhibitor cocktail tablet. The homogenate was mixed on a platform rocker and clarified by centrifugation. An aliquot of the resulting supernatant was combined with either anti-TSC2, anti-mTOR or anti-raptor antibody and immune complexes isolated with a goat anti-rabbit BioMag IgG (PerSeptive Diagnostics, Boston, MA) beads. The beads were collected, washed with CHAPS buffer, precipitated by centrifugation, and subjected to SDS-PAGE as described above. All blots were then developed with ECL and the autoradiographs were scanned for analysis as described above.

### Plasma concentrations of alcohol, glucose, amino acids and hormones

The plasma insulin concentration was measured using a commercial radioimmunoassay (RIA) for rat insulin (Linco Research, St. Charles, MO). Additionally, the plasma concentrations of total IGF-I, estradiol, and testosterone were determined using commercial RIA kits (DSLabs, Webster, TX). The plasma glucose and alcohol concentrations were determined by a rapid analyzer (Analox Instruments, Lunenburg, MA). Finally, the branched-chain amino acid concentrations were determined using reverse-phase HPLC after precolumn derivatization of amino acids with phenylisothiocyanate [24]. The plasma concentrations of glucose, insulin, IGF-I, estradiol, testosterone, branched-chain amino acids and alcohol were determined on blood collected immediately prior to injection of radiolabeled phenylalanine (i.e., time 0). Additionally, insulin and glucose were also determined on the blood sample collected 10 min after injection of phenylalanine. The original homeostasis model assessment (HOMA), defined as the [fasting insulin concentration (μU/ml) × glucose concentration (mmol/L)]/22.5, was used as an index of whole-body insulin resistance, as described by Matthews et al [25]. To better assure that a steady-state was achieved, glucose and insulin concentrations were determined at two time points. Because there was no difference in the glucose or insulin concentrations between the 0 time point and the 10-min time point (data not shown), these data were averaged for each rat and HOMA calculated using this average value. The advantages and disadvantages of HOMA for estimating insulin resistance have been reported [26].

### IGF system components

The concentration of free IGF-I was determined by centrifugal ultrafiltration, as originally described [27]. Briefly, samples were diluted 1:5 with Krebs-Ringer bicarbonate buffer (pH 7.4; with 5% BSA) and prefiltered through a 0.22 μm filter (Millex-GV, Millipore, Molsheim, France) to remove debris. The prefiltered samples were then added to Amicon YMT 30 membranes and MPS-1 supporting devices (Amicon Division, W. R. Grace, Co., Beverly MA) and centrifuged at 1500 rpm at 37°C for 100 min. The ultrafiltrate was collected from 40–100 min of centrifugation and used for the IGF-I RIA.

We have also developed a multi-probe template for a ribonuclease protection assay (RPA) for the detection of the most abundant IGF binding proteins (IGFBPs). Primer selection for rat genes of interest was determined with the help of Genefisher software [28]. The lengths of amplified regions were chosen to allow distinct resolution during electrophoretic separation. Primers were synthesized (IDT, Coralville, IA) with restriction sites for EcoRI or KpnI at the 5' end and with three extra bases at the extreme 5' end (Table 1). PCR was conducted using HotStarTaq DNA Polymerase (Qiagen, Valencia, CA) and rat total RNA reverse-transcribed with Superscript™ First-Strand Synthesis System for RT-PCR (Invitrogen, Carlsbad, CA). PCR products were phenol:chloroform extracted, ethanol precipitated, and sequentially digested with KpnI and EcoRI (Promega, Madison, WI). Digested products were gel-purified, re-extracted, and cloned into KpnI/EcoRI-digested pBluescript II SK+ (Stratagene, La Jolla, CA). Plasmid DNA was isolated with both QIAprep^R ^Spin Miniprep and Plasmid Maxi Kits (Qiagen). Plasmids with inserts were verified by sequencing in the Molecular Genetics Core Facility at the PSU College of Medicine. Final constructs were linearized with EcoRI, gel-purified, and quantitated spectrophotometrically.

**Table 1:** Primer sequences for the determination of IGF binding proteins (IGFBPs)

ALS	Forward 5'-*GCA GAA TTC *GGC TGC AGA AGC TGT ACC TGG A-3' Reverse 5'-*GCA GGT ACC *ATT CGA TTG TGG CCC AGC TGC A-3'
IGFBP-1	Forward 5'-*GCA GAA TTC *TGA GCT TGC CGA GAG CCC AGA-3' Reverse 5'-*GCA GGT ACC *AGA GCC CAG CTT CTC CAT CCA GA-3'
IGFBP-2	Forward 5'-*GCA GAA TTC *GGA GAA CCA TGT GGA CGG AAC CA-3' Reverse 5'-*GCA GGT ACC *CCC ACG CTG TCC ATT CAG AGA CA-3'
IGFBP-3	Forward 5'-*GCA GAA TTC *CTC CTC CGA GTC TAA GCG GGA GA-3' Reverse 5'-*GCA GGT ACC *CAG CGG TAT CTA CTG GCT CTG CA-3'
IGFBP-4	Forward 5'-*GCA GAA TTC *ATC ACA GGT GCC TGC AGA AGC A-3' Reverse 5'-*GCA GGT ACC *TGG AAG TTG CCG TTG CGG TCA-3'
IGFBP-5	Forward 5'-*GCA GAA TTC *CGA GTC ATC CCT GCA CCT GAG A-3' Reverse 5'-*GCA GGT ACC *CCA CGT TTG CGG CCA CGA GA-3'
L32	Forward 5'-*GCA GAA TTC *CGG CCT CTG GTG AAG CCC AA-3' Reverse 5'-GC*A GGT ACC *CTT CTC CGC ACC CTG TTG TCG-3'
GAPDH	Forward 5'-*GCA GAA TTC *CTG GCC AAG GTC ATC CAT GAC A-3' Reverse 5'-*GCA GGT ACC *GGG GCC ATC CAC AGT CTT CTG-3'

### RNA extraction and RPA

Total RNA was extracted from gastrocnemius and liver using TRI Reagent (Molecular Research Center, Cincinnati, OH) and the mRNA content was determined by RPA. An aliquot (2 μl) of template was prepared using T7 Polymerase with buffer (Fermentas, Hanover, MD), NTPs and tRNA (Sigma-Aldrich), RNaisin and DNase (Promega), and ^32^P-UTP (Amersham Biosciences, Piscatawy, NJ). Unless otherwise noted, the entire RPA procedure including labeling conditions, component concentrations, sample preparation, and gel electrophoresis was as published (BD Pharmingen, San Diego, CA). Hybridization buffer was 80% formamide and 20% stock buffer (200 mM Pipes, pH 6.4, 2 M NaCl, and 5 mM EDTA). Hybridization proceeded overnight at 56°C in a dry bath incubator (Fisher Scientific, Pittsburgh, PA) without the use of mineral oil. Samples were treated with RNAse A+T_1 _(Sigma) in 1× RNAse buffer (10 mM Tris-HCl pH 7.5, 5 mM EDTA, and 300 mM NaCl) followed by Proteinase K (Fisher Scientific) in 1× Proteinase K buffer (50 mM Tris, pH 8.0, 1 mM EDTA, 1% Tween-20). Following ethanol precipitation, samples were resuspended in 5 ml of loading buffer (98% formamide (v/v), 0.05% xylene cyanol (w/v), 0.05% bromphenol blue (w/v), and 10 mM EDTA). Polyacrylamide gels were run in an S3S Sequencing System (Owl Separation Systems, Portsmouth, NH), transferred to chromatography paper, and dried (FB GD 45 Gel Dryer, Fisher Scientific). Gels were exposed to a PhosphorImager screen (Molecular Dynamics Inc., Sunnyvale, CA). Data were visualized and analyzed using ImageQuant software (Version 5.2, Molecular Dynamics). Signal densities for mRNAs were analyzed in the linear range and normalized to L32 or GAPDH mRNA, which yield comparable results (data not shown).

### Statistical analysis

Experimental data for each condition are summarized as means ± SE where the number of animals in each treatment group is indicated in the legend to the figure or table. Statistical evaluation of the data was performed using ANOVA followed post-hoc by Student-Neuman-Keuls test when the interaction was significant. Differences between the groups were considered significant when *P *< 0.05.

## Results

### Plasma alcohol concentration

The concentration of alcohol in the blood 2.5 h after administration of ethanol averaged 265 ± 24 mg/dL in young rats administered 75 mmol/kg. Mature rats given the same amount of alcohol per kg body weight had blood alcohol levels which were 45% lower (*P *< 0.05) than young animals (154 ± 23 mg/dL; hereafter referred to as the low-dose group). In contrast, mature rats administered 90 mmol/kg alcohol (hereafter referred to as the high-dose group) had a blood alcohol level which was not different from young rats administered 75 mmol/kg of ethanol (281 ± 33 mg/dL; P = NS).

### Body and muscle weights

The body weight of the mature rats was 70% greater than animals in the young group (413 ± 13 g vs 239 ± 9 g; *P *< 0.05). Furthermore, the weight of the gastrocnemius was also significantly increased by approximately 60% in mature animals (428 ± 18 mg wet weight), compared to young rats (266 ± 6 mg). As a result of these changes, the gastrocnemius-to-body weight ratio was not significantly altered between the two age groups (data not shown), suggesting sarcopenia (e.g., age-associated muscle atrophy) had not yet developed in the mature animals. There was also no difference in either the total protein or total RNA content of the gastrocnemius between age groups (data not shown). Finally, because of the short time frame, acute alcohol intoxication did not alter gastrocnemius weight, protein content or RNA content in either young or mature rats (data not shown).

### Muscle protein synthesis

**Figure 1:**
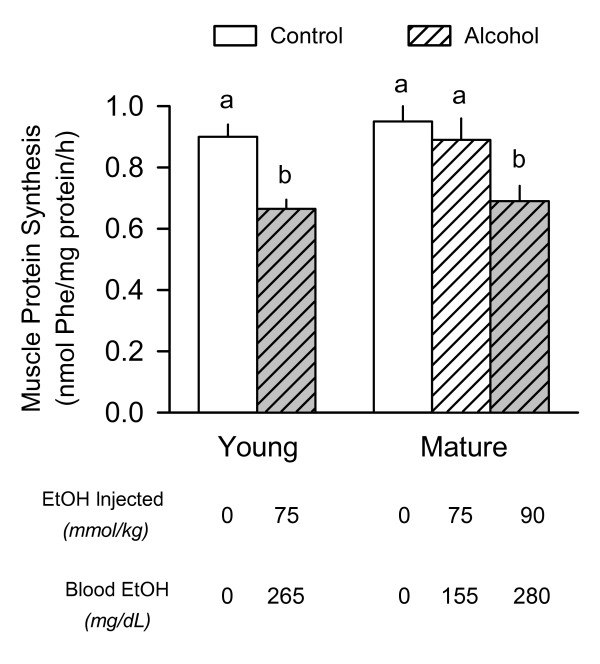
**Effect of acute alcohol intoxication on the *in vivo *rate of protein synthesis in skeletal muscle from young and mature rats**. Rats were injected intraperitoneally with either ethanol or saline (control) and protein synthesis in the fast-twitch gastrocnemius muscle was determined 2.5 h thereafter by incorporation of [^3^H]-phenylalanine (Phe) into protein. Two groups of mature rats were used in this study; rats in group 1 received the same amount of alcohol normalized to body weight as young rats (i.e., 75 mmol/kg), while rats in group 2 were injected with alcohol at a dose of 90 mmol/kg. The approximate blood alcohol concentration achieved in each of the three groups is presented at the bottom of the graph and the absolute values (means ± SEM) are presented in the text. Values for protein synthesis are means ± SEM where the sample size was 10, 10, 6, 7, 9, and 9 for the six groups, respectively. Values with different letters are significantly different from each other, *P *< 0.05. Values which share a common letter are not statistically different.

There was no difference in the basal rate of protein synthesis in the gastrocnemius removed from young and mature rats under control (i.e., no alcohol) conditions (Figure 1). Acute alcohol intoxication decreased in vivo determined muscle protein synthesis by 28% in young rats, compared to time- and age-matched controls. A comparable decrease in muscle protein synthesis was observed in the mature rats given the high-dose alcohol (90 mmol/kg) which produced blood alcohol levels comparable to those in young rats. In contrast, muscle protein synthesis was not decreased in mature adult rats given the low-dose of ethanol (75 mmol/kg), which produced a blood alcohol level only 65% of that detected in young rats given the same amount of alcohol.

### Phosphorylation of ribosomal protein (rp) S6

**Figure 2:**
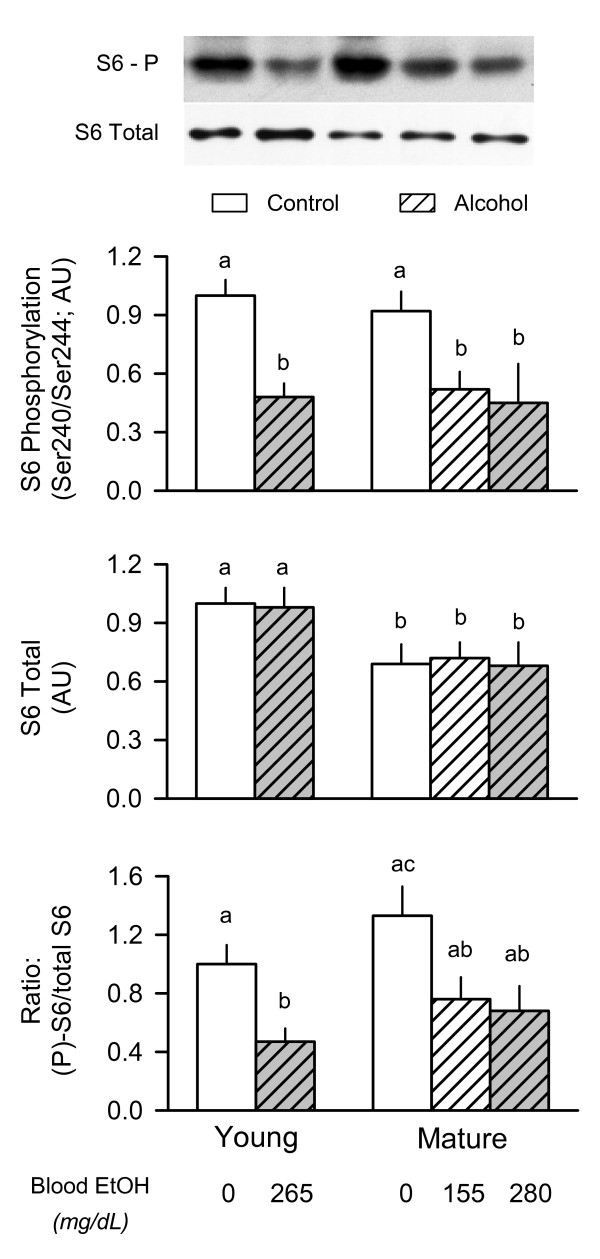
**Effect of acute alcohol intoxication on the total amount and phosphorylation of ribosomal protein S6 in skeletal muscle from young and mature rats**. Groups are the same as described in Figure 1. Gastrocnemius was collected 2.5 h after administration of alcohol or saline (control). *Insert at top: *representative Western blots of Ser240/Ser244-phosphorylated (P) S6 and total S6 protein in muscle. *Top and middle graphs: *densitometric analysis of immunoblots of Ser240/244-phosphorylated S6 and total S6, respectively. *Bottom graph*: ratio of phosphorylated (P) to total S6 protein in muscle. Values (means ± SEM) are expressed relative to the young saline-treated control group. Sample size was 10, 10, 6, 7, 9, and 9 for the six groups, respectively. Values with different letters are significantly different from each other, *P *< 0.05. Values which share a common letter are not statistically different.

The phosphorylation of S6K1 and 4E-BP1 is routinely use as surrogate markers of mTOR kinase activity [8]. However, under basal conditions in vivo, there is little constitutive Thr389-phosphorylation of S6K1 which is necessary for full activation of the enzyme. Therefore, we assessed the phosphorylation of rpS6 at Ser240/Ser244, a site specifically phosphorylated by S6K1 and S6K2 [29]. As illustrated in Figure 2 (middle graph), we detected a 30% decrease in the total amount of rpS6 in skeletal muscle of mature compared with young rats. Despite the reduction in total rpS6 in muscle from mature rats, the extent of S6 phosphorylation was not different between young and mature rats under basal conditions (Figure 2, top graph). Moreover, the alcohol-induced decrease in S6 phosphorylation was comparable between mature rats receiving either the low- or high-dose of ethanol. The ratio of the phosphorylated S6 to total S6 protein in muscle showed an alcohol-induced change comparable to that reported for S6 phosphorylation alone (Figure 2, bottom graph). Similar changes in Ser235/Ser236 phosphorylation, which represents an initiating phosphorylation event, were also observed in muscle from young and mature rats (data not shown).

### Phosphorylation of 4E-BP1 and formation of the eIF4F complex

**Figure 3:**
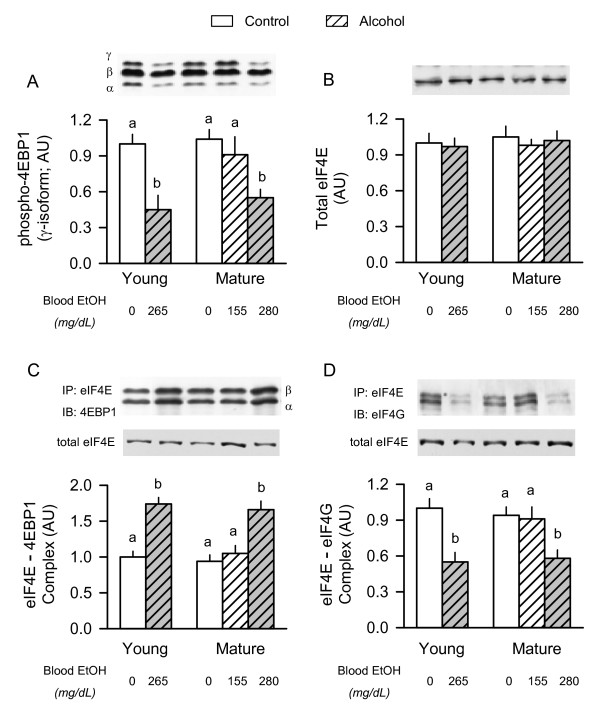
**Effect of acute alcohol intoxication on 4E-BP1 phosphorylation and the formation of the active eIF4F complex in skeletal muscle from young and mature rats**. Groups are the same as described in Figure 1. Gastrocnemius was collected 2.5 h after administration of alcohol or saline (control). *Insert above each graph: *representative Western blots. *Panels A and B: *densitometric analysis of immunoblots of γ-phosphorylated 4E-BP1 and total eIF4E in muscle homogenate, respectively, *Panels C and D*: eIF4E was immunoprecipitated (IP) and the amount of 4EBP1 and eIF4G bound to eIF4E was assessed by immunoblotting (IB). There was no age- or alcohol-induced change in total eIF4E in the immunoprecipitate (data not shown). In panel C, the α- and β-isoforms of 4E-BP1 are indicated. Values (means ± SEM) are expressed relative to the young saline-treated control group. Sample size was 10, 10, 6, 7, 9, and 9 for the six groups, respectively. Values with different letters are significantly different from each other, *P *< 0.05. Values which share a common letter are not statistically different.

4E-BP1 is phosphorylated in a hierarchical manner and the various phosphorylated forms of the protein are resolved into three bands which are designated α-, β-, and γ [30]. In contrast to the hypo-phosphorylated α- and β-isoforms, the γ-isoform is highly phosphorylated and does not bind eIF4E. There was no difference in either the total amount of 4E-BP1 (data not shown) or the amount of γ-phosphorylated 4E-BP1 in gastrocnemius of young and mature rats (Figure 3A). Acute alcohol intoxication reduced 4E-BP1 γ-phosphorylation by 60% in young rats. A comparable decrease was seen in mature rats administered the high-dose of alcohol, but not in mature animals given the low-dose of alcohol.

Western blot analysis of tissue homogenates showed no age- or alcohol-induced change in the amount of total eIF4E (Figure 3B). However, the function of eIF4E can also be controlled by its binding to a family of cap-dependent translational repressors, of which 4E-BP1 is the most prominent family member in skeletal muscle. Hyperphosphorylation of 4E-BP1 liberates it from eIF4E and allows binding of eIF4E with eIF4G and the stimulation of protein synthesis. There was no difference in the amount of the eIF4E·4EBP1 complex or the eIF4E·eIF4G complex in gastrocnemius between young and mature rats under basal conditions (Figure 3C and 3D). Acute alcohol intoxication resulted in a redistribution of eIF4E from the active to the inactive complex in young rats and mature rats given the high-dose of alcohol. In contrast, no such redistribution of eIF4E was detected in mature rats given the low-dose of alcohol. These alcohol-induced changes in the distribution of eIF4E were independent of a change in the eIF4E content in the immunoprecipitate (see representative blot, Figure 3C and 3D).

### eIF4G phosphorylation

**Figure 4:**
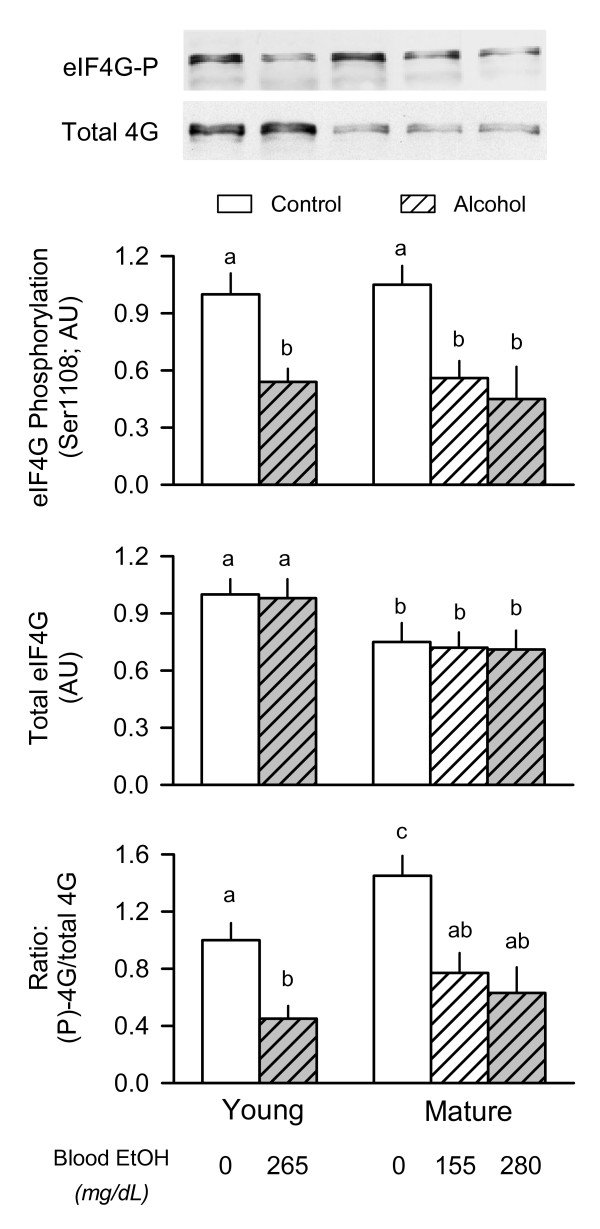
**Effect of acute alcohol intoxication on the total amount and phosphorylation of eIF4G in skeletal muscle from young and mature rats**. Groups are the same as described in Figure 1. Gastrocnemius was collected 2.5 h after administration of alcohol or saline (control). *Inset at top: *representative Western blots of phosphorylated and total eIF4G. *Top and middle graphs: *densitometric analysis of immunoblots of Ser1108-phosphorylated eIF4G and total eIF4G, respectively, in muscle. *Bottom graph: *ratio of phosphorylated (P) to total eIF4G in muscle. Values (means ± SEM) are expressed relative to the young saline-treated control group. Sample size was 10, 10, 6, 7, 9, and 9 for the six groups, respectively. Values with different letters are significantly different from each other, *P *< 0.05. Values which share a common letter are not statistically different.

The interaction between eIF4E and eIF4G can also be regulated by the phosphorylation of eIF4G which is enhanced by mitogens and decreased by the mTOR inhibitor rapaymcin [31]. The total amount of eIF4G in the muscle homogenate was reduced approximately 30% in mature rats, compared to young adult animals, but there was no alcohol effect on total eIF4G (Figure 4, middle graph). In contrast, the relative amount of constitutive eIF4G Ser1108-phosphorylation was similar in young and mature rats, but acute alcohol intoxication decreased eIF4G phosphorylation 45–50% in all groups regardless of age or the alcohol dose administered (Figure 4 top graph). As a result of these changes, the ratio of phosphorylated to total eIF4G was increased under basal conditions in mature rats compared to young animals, but alcohol administration decreased the ratio in both groups regardless of animal age (Figure 4, bottom graph).

### mTOR complex 1

**Figure 5:**
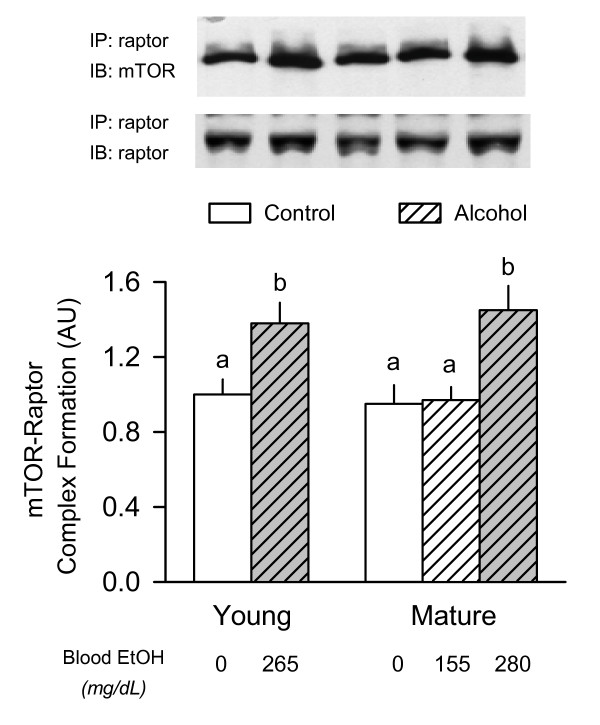
**Effect of acute alcohol intoxication on the binding of mTOR with raptor in skeletal muscle from young and mature rats**. Groups are the same as described in Figure 1. Gastrocnemius was collected 2.5 h after administration of alcohol or saline (control). *Inset at top: *raptor was immunoprecipitated (IP) and the amount of mTOR and raptor determined by immunoblotting (IB). *Bottom graph: *densitometric analysis of immunoblots of mTOR bound to immunoprecipitated raptor in muscle. There was no difference among the various groups for the amount of immunoprecipitated raptor (mean data not shown). Values (means ± SEM) are expressed relative to the young saline-treated control group. Sample size was 10, 10, 6, 7, 9, and 9 for the six groups, respectively. Values with different letters are significantly different from each other, *P *< 0.05. Values which share a common letter are not statistically different.

As a result of its predominant role in regulating mTOR activity, the amount of the mTOR complex 1 (mTORC1) was determined. The mTORC1 is composed of at least four proteins, including mTOR, raptor, GβL, and PRAS40 [32]. Western blot analysis of whole muscle homogenate did not demonstrate a significant age- or alcohol-induced change for total mTOR, raptor, GβL or PRAS40 (data not shown). However, in muscle from either alcohol-treated young rats or mature rats given the high-dose of alcohol, the association of mTOR with immunoprecipitated raptor was increased approximately 35% (Figure 5). Such a change in mTOR·raptor binding was not seen in muscle of mature rats given the lower dose of alcohol. In contrast, there was no age-or alcohol-induced change in the binding of GβL or PRAS40 to raptor (data not shown).

### TSC and AMPK

mTOR activity is regulated at least in part by the phosphorylation of TSC2 and/or the dimerization of TSC2 with TSC1, which can be modulated independently by insulin and nutrients [33]. However, by Western blot analysis there was no significant age- or alcohol-induced change in either the total amount of TSC1 and TSC2, or the association of TSC1 with TSC2 (data not shown). Although activation of the energy sensor AMPK decreases protein synthesis in muscle via a mTOR-dependent mechanism [34], we detected no age- or alcohol-induced change in either the total amount or Thr172-phosphorylated AMPK (data not shown).

### IGF system

**Figure 6:**
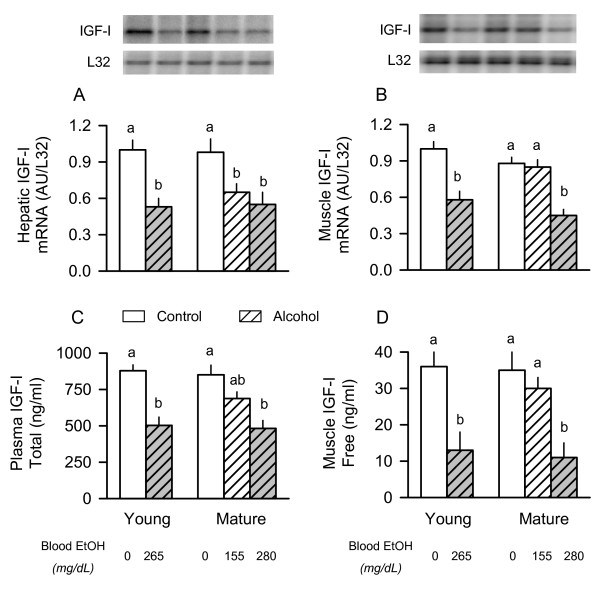
**Effect of acute alcohol intoxication on IGF-I concentration and mRNA content in young and mature rats**. *Inset: *representative autoradiographs from a nuclease protection assay (RPA) for IGF-I in liver and gastrocnemius. *Graphs A and B: *densitometric analysis of RPAs performed on liver and muscle, respectively. *Graphs C and D: *plasma and muscle IGF-I peptide concentrations were determined by RIA. Groups are the same as described in Figure 1. Values (means ± SEM) where the sample size was 10, 10, 6, 7, 9, and 9 for the six groups, respectively. Values with different letters are significantly different from each other, *P *< 0.05. Values which share a common letter are not statistically different.

Adequate IGF-I is necessary for maintenance and accretion of lean body mass, and previous studies reported a strong correlation between muscle IGF-I and protein synthesis in response to chronic alcohol consumption and in other catabolic conditions [5,35]. Therefore, the IGF-I mRNA content of tissues (liver and muscle) and the IGF-I concentration in blood and muscle was determined. There was no age-dependent change in the IGF-I mRNA content of either liver or gastrocnemius under basal conditions (Figure 6A and 6B). Alcohol acutely decreased the hepatic IGF-I mRNA content in all groups of rats regardless of age or, for the old animals, the amount of alcohol administered (Figure 6A). In contrast, alcohol decreased IGF-I mRNA content in muscle only in young rats and mature animals given the high-dose of ethanol (Figure 6B). No such decrease in IGF-I mRNA was detected in muscle of the mature rats given the low-dose of alcohol.

While there was no age-dependent change in the plasma total IGF-I concentration under basal conditions, acute alcohol intoxication decreased plasma IGF-I to a similar extent (40–45%) in both young and mature rats given high-dose alcohol (Figure 6C). Mature rats given the lower dose of alcohol had a total IGF-I concentration intermediate between two other groups of mature rats. Finally, the concentration of free IGF-I, which is the biologically active form of the hormone, was assessed in skeletal muscle. Although there was no age-dependent change in the basal concentration of free IGF-I in muscle, the concentration of this anabolic hormone was markedly reduced in both young rats administered alcohol and in mature rats given the high-dose of ethanol (Figure 6D).

The hepatic mRNA content for the various IGFBPs provides a surrogate marker for their circulating concentration [36], which can influence the bioavailability and bioactivity of IGF-I [37]. The effect of acute alcohol intoxication on IGFBP mRNA expression in liver from young and old rats is presented in Table 2. The most striking change noted in the current study was related to IGFBP-1. In young rats, acute alcohol intoxication increased hepatic IGFBP-1 mRNA nearly 80%. While in mature rats given the high-dose of alcohol, IGFBP-1 mRNA expression was increased even further. Such an alcohol-induced stimulation of IGFBP-1 was not seen in mature rats administered the lower dose of alcohol. There was no significant age- or alcohol-induced effect on IGFBP-3 or IGFBP-4 mRNA content in liver. In contrast, under basal conditions IGFBP-2 mRNA was reduced by approximately 50% in mature rats, compared to young animals. Furthermore, alcohol decreased IGFBP-2 mRNA in young rats, while no such alcohol-induced decrease in IGFBP-2 was detected in mature animals. The large majority of circulating IGF-I is carried in a ternary complex consisting of IGF-I, IGFBP-3 and the acid labile subunit (ALS). Again, under basal conditions there was no age-dependent change in hepatic ALS mRNA, but alcohol decreased ALS expression in young rats and mature animals given high-dose ethanol.

**Table 2:** Effect of acute alcohol intoxication on liver IGFBP mRNA content in young and mature adult male F344 rats

	Young		Mature – Low Dose EtOH*		Mature – High Dose EtOH	
	Control	Alcohol	Control	Alcohol	Control	Alcohol
IGFBP-1, *AU/L32*	1.00 ± 0.08^a^	1.78 ± 0.22^b^	0.91 ± 0.17^a^	1.21 ± 0.19^a^	0.94 ± 0.09^a^	2.68 ± 0.26^c^
IGFBP-2, *AU/L32*	1.00 ± 0.08^a^	0.64 ± 0.05^b^	0.45 ± 0.08^b^	0.52 ± 0.05^b^	0.47 ± 0.06^b^	0.49 ± 0.09^b^
IGFBP-3, *AU/L32*	1.00 ± 0.11	1.05 ± 0.15	0.87 ± 0.11	0.79 ± 0.09	0.84 ± 0.08	0.78 ± 0.14
IGFBP-4, *AU/L32*	1.00 ± 0.07	1.18 ± 0.12	0.90 ± 0.08	0.97 ± 0.07	0.89 ± 0.11	0.95 ± 0.09
ALS, *AU/L32*	1.00 ± 0.09^a^	0.68 ± 0.07^b^	1.11 ± 0.11^a^	1.01 ± 0.11^a^	1.08 ± 0.09^a^	0.61 ± 0.07^b^

Values are means ± SEM where the sample size was 10, 10, 6, 7, 9, and 9 for the six groups, respectively. *Mature rats were administered either "low" dose alcohol (75 mmol/kg) or "high" dose alcohol (90 mmol/kg); young rats were injected with 75 mmol/kg. Abundance of IGFBP-5 was below the detection limit for liver in all animals and data are not reported. Values with different letters for a particular hormone are significantly different from each other, *P *< 0.05. Values with the same letter are not significantly different.

We also quantitated the mRNA content of IGFBPs in skeletal muscle because changes in these proteins can affect the bioavailability of IGF-I in this tissue and thereby indirectly modulate protein synthesis [38,39]. IGFBP-1, IGFBP-2, and ALS mRNA were not reliably detected by RPA in skeletal muscle (data not shown). IGFBP-3 and IGFBP-4 showed no age- or alcohol-induced changes in muscle (Table 3). In contrast, IGFBP-5 mRNA content was decreased in young rats by alcohol. Mature rats also showed an alcohol-induced decrease muscle IGFBP-5 mRNA in response to high-dose but not low-dose alcohol.

**Table 3:** Effect of acute alcohol intoxication on muscle IGFBP mRNA content in young and mature adult male F344 rats

	Young		Mature – Low Dose EtOH*		Mature – High Dose EtOH	
	Control	Alcohol	Control	Alcohol	Control	Alcohol
IGFBP-3, *AU/L32*	1.00 ± 0.08	0.87 ± 0.11	0.81 ± 0.12	0.83 ± 0.11	0.79 ± 0.11	0.77 ± 0.15
IGFBP-4, *AU/L32*	1.00 ± 0.12	0.95 ± 0.12	1.24 ± 0.21	1.18 ± 0.19	1.14 ± 0.12	1.13 ± 0.14
IGFBP-5, *AU/L32*	1.00 ± 0.06^a^	0.66 ± 0.06^b^	0.84 ± 0.07^a^	0.85 ± 0.06^ab^	0.89 ± 0.08^a^	0.49 ± 0.07^b^

Values are means ± SEM where the sample size was 10, 10, 6, 7, 9, and 9 for the six groups, respectively. *Mature rats were administered either "low" dose alcohol (75 mmol/kg) or "high" dose alcohol (90 mmol/kg); young rats were injected with 75 mmol/kg. The abundance of IGFBP-1, BP-2 and ALS was below the detection limit in muscle for all rats and data are not reported. Values with different letters for a particular hormone are significantly different from each other, *P *< 0.05. Values with the same letter are not significantly different.

### Plasma hormone and substrate concentrations

Alterations in the milieu of other hormones can also regulate muscle protein synthesis and were quantitated in an attempt to include or exclude potential mediators (Table 4). In this regard, the basal plasma insulin concentration was elevated approximately 2-fold in both groups of mature rats compared to values in young adult rats. However, the plasma glucose concentration was not statistically altered in young versus mature rats under basal conditions. As a result, under basal conditions the mature rats were determined to be insulin resistant as evidenced by the increased HOMA. Alcohol tended to decrease the insulin concentration in all groups, but these changes failed to achieve statistical significance. In contrast, acute alcohol intoxication produced a significant hyperglycemic effect in young rats as well as in mature rats given high-dose of alcohol. Therefore, while alcohol tended to increase HOMA in all three experimental groups, these changes failed to achieve statistical significance. Hence, the two doses of alcohol administered to the mature rats did not appear to differentially affect insulin sensitivity. Sex hormones may also influence protein balance. Our data indicate there was no age-dependent effect on the basal plasma testosterone concentration and that alcohol acutely decreased testosterone in all three groups to a comparable extent. Furthermore, we detected no consistent age- or alcohol-induced change in the estradiol concentration among the various treatment groups. Finally, the plasma concentrations of the three branched-chain amino acids were determined because these amino acids are primarily responsible for acutely regulating muscle protein synthesis [40]. There was no difference in the basal concentration of leucine, isoleucine or valine between young and mature rats. However, acute alcohol intoxication did significantly increase the leucine concentration in young and mature rats given the high dose of alcohol (44% and 48%, respectively). No such alcohol-induced increase was observed in mature rats given the low dose of alcohol. Similarly, alcohol tended to increase the plasma concentrations of both isoleucine and valine in young and mature rats given high dose alcohol, but the change failed to achieve statistical significance.

**Table 4:** Effect of acute alcohol intoxication on plasma hormone and metabolic substrate concentrations in young and mature adult male F344 rats

	Young		Mature – Low Dose EtOH*		Mature – High Dose EtOH	
	Control	Alcohol	Control	Alcohol	Control	Alcohol

Insulin, *ng/ml*	1.09 ± .09^a^	0.84 ± .13^a^	2.13 ± .25^b^	2.03 ± .21^b^	2.12 ± .33^b^	2.09 ± .24^b^

Testosterone, *ng/ml*	2.8 ± 0.2^a^	1.6 ± 0.2^b^	2.7 ± 0.2^a^	1.3 ± 0.2^b^	2.5 ± 0.3^a^	1.5 ± 0.3^b^

Estradiol, *pg/ml*	2.7 ± 0.5	2.1 ± 0.5	3.5 ± 0.4	2.9 ± 0.4	3.6 ± 1.2	2.7 ± 0.4

Glucose, *mmol/L*	7.0 ± 0.5^a^	11.2 ± 1.1^b^	8.4 ± 0.5^a^	8.7 ± 0.4^a^	8.5 ± 0.5^a^	10.8 ± 0.8^ab^

HOMA	8.3 ± 0.8^a^	10.3 ± 1.3^a^	19.6 ± 3.3^b^	21.6 ± 3.1^b^	19.6 ± 3.4^b^	23.7 ± 4.3^b^

Leucine, μmol/L	95 ± 6^a^	137 ± 11^b^	97 ± 4^a^	107 ± 10^a^	95 ± 6^a^	141 ± 12^b^

Isoleucine, μmol/L	81 ± 10	104 ± 11	85 ± 7	91 ± 11	86 ± 8	116 ± 9

Valine, μmol/L	138 ± 12	166 ± 21	127 ± 15	134 ± 12	133 ± 14	155 ± 12

Values are means ± SEM where the sample size was 10, 10, 6, 7, 9, and 9 for the six groups, respectively. *Mature rats were administered either "low" dose alcohol (75 mmol/kg) or "high" dose alcohol (90 mmol/kg); young rats were injected with 75 mmol/kg. Blood was obtained from overnight fasted rats immediately prior to determination of muscle protein synthesis (e.g., time 0). Glucose and insulin concentrations were determined at time 0 and 10 min after injection of phenylalanine, and values averaged because there was no statistical difference between time points (data not shown). Values with different letters for a particular hormone are significantly different from each other, *P *< 0.05. Values with the same letter are not significantly different. HOMA = homeostasis model assessment for insulin resistance which is calculated as reported by Matthews et al [25].

## Discussion

Results from the present study indicate muscle protein synthesis in young adult and mature F344 rats is equally sensitive to the suppressive effect of acute alcohol intoxication. However, this effect was only equivalent when the amount of alcohol administered to mature rats was increased in order to produce a blood alcohol level comparable to that seen in young rats. Under conditions where the young and mature rats were administered the same amount of alcohol (75 mmol/kg), the blood alcohol concentration was 40% lower in the mature rats. The lower concentration of alcohol in the older mature rats is consistent with previous in vivo studies in Fischer 344 rats which reported an increased ethanol elimination in animals naive to alcohol [41,42]. Therefore, the alcohol-induced decrease in muscle protein synthesis produced by acute intoxication is not limited to rapidly growing animals as long as comparisons between different aged rats are matched to the prevailing blood alcohol level and not the dose of alcohol administered.

The mature rats used in the present study should not be categorized as "aged" animals. We purposefully avoided the use of aged rats (e.g., > 18 months of age) because of the potential for sacropenia and increased susceptibility to alcohol toxicity [42]. In this regard, the muscle weight-to-body weight ratios and the rates of muscle protein synthesis were not different between young and mature rats in our study. Hence, these data suggest that overt sacropenia had not yet developed in mature rats and that muscle protein synthesis, at least after an overnight fast, is relatively unchanged. These data are internally consistent with the amount and phosphorylation (i.e., activation) of several protein factors known to regulate mRNA translation. For example, there was no difference in either the total amount or phosphorylation state for 4E-BP1 and the amount of the active eIF4E·eIF4G complex in muscle from young and mature rats under basal fasted conditions. While we did detect a reduction in the total amount of both rpS6 and eIF4G between young and mature rats, the amount of these proteins phosphorylated remained unchanged (e.g. S6) or was even increased (e.g., eIF4G) in young rats compared with mature animals.

**Figure 7:**
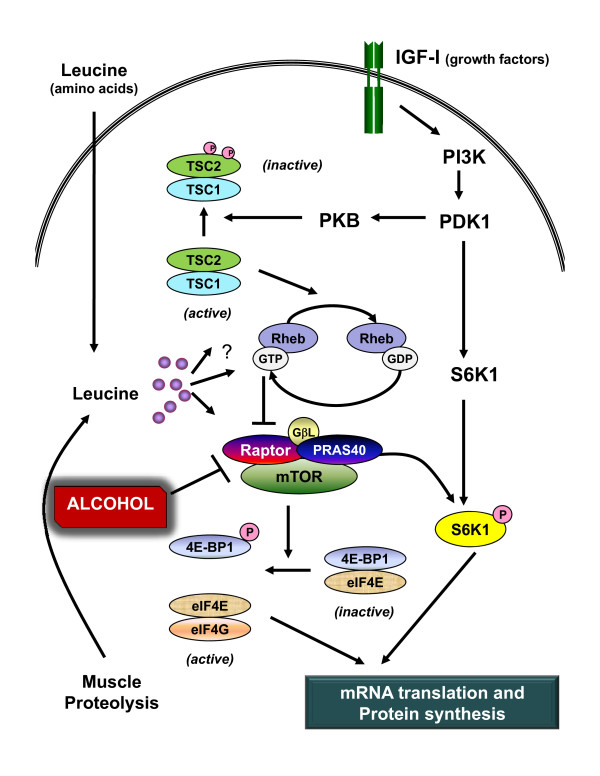
**Schematic of possible site for alcohol-induced inhibition of muscle protein synthesis**. This paradigm illustrates the central role served by mTOR (mammalian target of rapamycin) at integrating the independent anabolic signals generated by IGF (insulin-like growth factor-I and the amino acid leucine. mTOR is present in a multimeric protein complex termed mTORC1 which consists of itself, raptor, PRAS40 (proline-rich Akt substrate) and GβL (G protein β-subunit-like protein/mLST8). In addition, a second protein complex exists (mTORC2) consisting of mTOR, GβL, rictor, and mSin1 (mammalian stress-activated protein kinase-interacting protein) and this complex is not shown because it does not appear to directly modulate protein synthesis. mTORC1 has kinase activity which phosphorylates both S6K (ribosomal protein S6 kinase)-1 and 4E-BP1 [eukarytoic initiation factor (eIF) 4E-binding protein-1]. The phosphorylation of 4E-BP1 results in the redistribution of eIF4E from an inactive eIF4E-4EBP1 complex to the active eIF4E-eIF4G complex which, along with other proteins, forms the functional eIF4F complex and stimulates mRNA translation. Present data indicates acute alcohol intoxication increases the association of mTOR and raptor suggesting a "closed" inhibitory confirmation which is consistent with the alcohol-induced decrease in 4E-BP1 and S6K1 phosphorylation. Other reports [6] indicate alcohol does not alter phosphorylation of the IGF-I receptor or protein kinase B (PKB; aka Akt), or the formation of the tuberous sclerosis complex (TSC)-1/TSC-2. Although several possible mechanisms have been reported [51,52], the signal transduction pathway used by leucine to stimulate protein synthesis in skeletal muscle is poorly defined but is independent of PKB activation. Solid lines with arrow heads represent stimulation, whereas lines terminating in a perpendicular line represent inhibition. Numerous proteins and cofactors have been omitted from the diagram to simplify and improve clarity.

As indicated above, the rate of protein synthesis in gastrocnemius was similarly reduced in young and mature rats when the prevailing blood alcohol level was matched. Because the RNA content in muscle was not altered by alcohol, this decreased muscle protein synthesis indicates a concomitant reduction in translational efficiency. Furthermore, the differential effect of the two doses of alcohol in mature rats permits the exclusion of potential underlying mechanisms for the reduction in protein synthesis. For example, mature rats which received either dose of alcohol had a similar decrease in both the phosphorylation of rpS6 and eIF4G. As muscle protein synthesis per se was only reduced in the high-dose alcohol group, this suggests an alcohol-induced change in the activity of S6K1 (the upstream kinase responsible for rpS6) is an unlikely mediator of this catabolic effect. In contrast, the differential effect of low- and high-dose alcohol on protein synthesis was closely paralleled by the decreased phosphorylation of 4E-BP1 as well as the increased formation of the inactive eIF4E·4EBP1 complex and decreased amount of the eIF4E·eIF4G complex. This alcohol-induced decrease in the binding of eIF4E with eIF4G and the subsequent reduction in the functional eIF4F complex would be expected to limit protein synthesis at the step involving binding of mRNA to the 43S preinitiation complex [8]. A schematic representation of the effect of alcohol on mTOR-mediated signal transduction is presented in Figure 7.

Our data clearly demonstrate acute alcohol intoxication increases the association of mTOR bound to raptor. These findings are consistent with data from other in vitro studies using myocytes where a reduction in protein synthesis produced by amino acid or leucine deprivation was associated with an increase in mTOR·raptor formation [34]. Collectively, these data are supportive of alcohol impairing mTOR kinase activity by promoting a "closed conformation" which has been proposed to render it less active [43]. This alcohol-induced change in mTOR·raptor also appears to be mediated by a mechanism which is AMPK- and TSC-independent, although TSC activity per se still needs to be directly assessed in response to alcohol. The mTORC1 complex also binds PRAS40 and GβL via its interaction with raptor [44,45]; however, there was no detectable change in the total amount or the binding of these two substrates to raptor in response to alcohol. Finally, a decrease in the intracellular leucine concentration might also contribute to the increased mTOR· raptor association, although this possibility seems less likely because the plasma concentration of leucine was elevated in alcohol-treated rats compared to control animals and alcohol does not increase muscle protein degradation [46].

The prevailing circulating concentration of various hormones or the ability of tissues to respond to these agents can markedly influence muscle protein synthesis. In this regard, our studies provide evidence supporting the importance or lack there of for several hormones. For example, testosterone is a potent anabolic agent capable of increasing protein synthesis and the accretion of lean body mass [47]. However, because alcohol decreased testosterone to the same extent in all three groups, it seems unlikely that a differential regulation of this anabolic hormone is solely responsible for the alcohol-induced decrease in muscle protein synthesis. Similarly, there was no significant alcohol effect on plasma estradiol in any of the three groups, functionally excluding changes in this hormone as a mediator for the decrease in protein synthesis. Changes in the prevailing plasma concentration of insulin can lead to proportional changes in muscle protein synthesis [48]. However, acute alcohol intoxication did not significantly decrease the circulating insulin concentration in any group. Moreover, we calculated an index of insulin resistance (e.g., HOMA) and found that although the mature rats as a group were insulin resistant compare to young animals, there was no difference between mature rats that received either the low- or high-dose of ethanol. Collectively, these data argue against a change in either insulin concentration and/or insulin action as a causative mechanism for the alcohol-induced decrease in muscle protein synthesis. Finally, there was no difference in the plasma concentrations for the three branched-chain amino acids – leucine, isoleucine and valine – between young and mature rats under basal control conditions. Moreover, acute alcohol intoxication produced a comparable increase in the plasma leucine concentration between young rats and mature animals given the high dose of alcohol, and a similar trend was observed for isoleucine and valine. In addition, acute alcohol intoxication does not appear to alter the plasma concentration of total amino acids [24]. Overall, although these data suggest that an acute reduction in the circulating concentration of amino acids in general and branched-chain amino acids in particular is not responsible for the alcohol-induced decrease in muscle protein synthesis, we cannot exclude the possibility that alcohol impairs translation by decreasing the intracellular leucine concentration.

In contrast, although we cannot exclude the possibility that circulating IGF-I mediates the reduction in muscle protein synthesis in response to alcohol, our data demonstrate a close association between the content of IGF-I mRNA and IGF-I protein in muscle and changes in protein synthesis within the same muscle. This is the first report of the concentration of free bioavailable IGF-I in muscle in response to acute alcohol intoxication. The alcohol-induced decrease in free IGF-I within muscle is likely the result of a reduction in the synthesis of IGF-I by muscle as well as the suspected rise in the plasma concentration of IGFBP-1 which is known to sequester free IGF-I. Moreover, elevations in circulating IGFBP-1 have been shown to decrease muscle protein synthesis under both in vivo and in vitro conditions [39]. Although plasma IGFBP-1 concentrations were not directly assessed in the current study, hepatic IGFBP-1 mRNA content is a reliable surrogate marker for this particular binding protein. Finally, alcohol-induced changes in IGFBP-5 mRNA content in skeletal muscle were also directly proportional to changes in IGF-I and protein synthesis. This decrease in muscle IGFBP-5 is consistent with the reduction seen in several other catabolic conditions with accompanying muscle wasting [49]. Because changes in IGF-I produce proportional changes in IGFBP-5 in cultured myocytes [50], the observed reduction in IGFBP-5 in response to alcohol may occur secondary to the reduction in muscle IGF-I. Hence, the reduction in muscle IGF-I is not caused by the decrease in IGFBP-5 but is instead the mechanism for the reduction in this particular IGFBP. Overall, the mechanism by which the alcohol-induced decrease in autocrine/paracrine produced IGF-I inhibits muscle protein synthesis remains to be determined. Although previous studies have reported acute alcohol does not alter constitutive IGF-I or insulin receptor tyrosine phosphorylation or Thr-308 phosphorylation of Akt [6], the kinase activity per se of these proteins has not been directly assessed. Hence, it remains possible that alcohol decreases mRNA translation and protein synthesis by impairing IGF-I signal transduction directed via a TSC-independent mechanism. Alternatively, the alcohol-induced decrease in muscle IGF-I may be associated with but not causally related to the reduction in muscle protein synthesis.

## Conclusion

Our data indicate that young and mature adult male rats demonstrate the same reduction in muscle protein synthesis when blood alcohol levels are closely matched but, because of the apparently greater rate of ethanol clearance in adult male rats, this requires mature rats be administered a relatively larger dose of alcohol. The differential response observed in the mature rats to the low- and high-dose alcohol suggests that changes in 4E-BP1 phosphorylation, the distribution of eIF4E between active and inactive eIF4F complexes, and the increased association of mTOR and raptor mediates the alcohol-induced decrease in mRNA translation. These changes in translation and protein synthesis in skeletal muscle in response to acute alcohol intoxication were independent of changes in plasma testosterone, estradiol, insulin, and branched-chain amino acids but were associated with the reduction in free muscle IGF-I peptide. Moreover, this potential cellular mechanism by which alcohol inhibits muscle protein synthesis was seen in both young and adult male rats.

## Declaration of competing interests

The authors declare that they have no competing interests.

## Authors' contributions

CHL helped design the study, participated in the experimental protocol, analyzed data, and wrote the manuscript. AMP participated in the experimental protocol and performed all Western blots. GJN designed the nested primers used for the analysis of the various IGFBPs and ran the subsequent RPA analysis. TCV helped design the study, participated in the experimental protocol and analyzed data related to muscle protein synthesis.
